# Comparison of tisagenlecleucel with conventional treatments for relapsed/refractory diffuse large B-cell lymphomas: a retrospective external comparator study

**DOI:** 10.1038/s41408-023-00889-5

**Published:** 2023-08-18

**Authors:** Sohee Park, Ju Hwan Kim, Songhee Kim, Jisu Kang, Seoyoung Moon, Seok Jin Kim, Ju-Young Shin

**Affiliations:** 1 Sungkyunkwan University School of Pharmacy, Seoul, South Korea; 2 Research Department of Practice and Policy, School of Pharmacy, University College London, London, UK; 3 Research Institute for Future Medicine, Samsung Medical Center, Seoul, South Korea; 4 Department of Medicine, Samsung Medical Center, Sungkyunkwan University School of Medicine, Seoul, South Korea; 5 Department of Biohealth Regulatory Science, Sungkyunkwan University, Seoul, South Korea; 6 Department of Clinical Research Design & Evaluation, Samsung Advanced Institute for Health Sciences & Technology (SAIHST), Sungkyunkwan University, Seoul, South Korea

**Keywords:** Epidemiology, Targeted therapies, Chemotherapy

## Introduction

Tisagenlecleucel (tisa-cel) is an anti-CD19 chimeric antigen receptor T-cell (CAR-T) therapy first approved by the US food and drug administration in 2017 for relapsed or refractory diffuse large B-cell lymphoma (r/r DLBCL) after at least two treatment lines based on the efficacy observed in the JULIET phase 2 single-arm trial (Study of Efficacy and Safety of CTL019 in Adult DLBCL Patients) [1]. Before the introduction of CAR-T therapies, pooled data analysis reported poor objective response rate of 26% and median overall survival (OS) of 6.3 months among patients with r/r DLBCL [2], which indeed appears to be inferior to the 52% and 12 months, respectively, observed in the JULIET study [3]. However, there is a scant data on head-to-head comparison between tisa-cel vs conventional treatments, and only one study by Maziarz et al. to date has demonstrated improved patient survival with tisa-cel using two individual patient data (IPD) from the JULIET study and historical treatment arm [4].

There is a growing interest to the use of externally derived real-world data as a proxy for control group to aid in interpreting the findings from single arm trial. In the case of Maziarz et al., the investigators had access to the IPD of both JULIET and historical control arm, enabling for regression or propensity score analyses to mitigate cross-trial differences. However, access to the IPD for both arms is not always possible, and use of published summary data for one of the arms to generate evidence on the comparative effectiveness may be inevitable. In this regard, we aimed to generate comparative effectiveness data with the combined uses of the published summary data and IPD derived from a routine care setting. Specifically, we explored OS associated with tisa-cel by comparing published summary data from JULIET study (NCT#02445248) and IPD from Samsung Medical Center-Lymphoma Cohort study (SMC-LCS; NCT#00822731 and NCT#01877109) [3, 5–7].

## Materials/subjects and methods

The real-world patient-level data were derived from two prospective cohort studies. The first SMC-LCS (2008–2011, NCT#00822731) and second SMC-LCS (2012–2017, NCT#01877109) enrolled patients who were diagnosed with Hodgkin and Non-Hodgkin lymphoma from September 2008 to February 2017, with the aim of developing prediction models for progression and outcomes of lymphoma, including DLBCL, in South Korea [5–7]. SMC-LCS evaluated all risk factors including diagnostic, treatment, and pre-treatment information at diagnosis. The study registry data contains longitudinal, de-identified, patient-level structured data and variables extracted from unstructured data through trained human curators following standardized policies and procedures.

To assess the treatment effect of tisa-cel, we compared full analysis set (FAS) of JULIET study with an external comparator group that included patients diagnosed with r/r DLBCL among SMC-LCS population. To improve comparability between the two data, patients in SMC-LCS were selected based on the eligibility criteria of JULIET **(**Table S1**)**. In addition, patients were required to have received at least third-line therapy as an index treatment, and excluded if they were outside the age range of JULIET study (22–76 years) at index treatment or had missing value for the variables included for computing propensity score weights.

OS was compared between tisa-cel and conventional treatments. For the tisa-cel group, OS was assessed from the time of infusion. Kaplan–Meier (KM) curve for OS in JULIET FAS was informed from Schuster et al. (Figure 3 Panel D in that publication) [3], and reconstructed using Engauge Digitizer software (version 12.1) [8] and the approach of Guyot et al. [9]. For conventional treatment group, OS was defined as the time interval from index treatment initiation to the date of death due to any reason or last follow-up visit. Index treatment (i.e., conventional treatment) in a routine care setting at SMC included ICE regimen (ifosfamide, carboplatin, etoposide plus dexamethasone) or GDP regimen (gemcitabine, dexamethasone, cisplatin).

Propensity score weighting using matching-adjusted indirect comparison (MAIC) approach was used to control for potential confounders [10]. The following variables available both in JULIET and SMC-LCS without any missing values were adjusted for in the MAIC: age, Eastern Cooperative Oncology Group (ECOG) performance, disease stage at study entry, number of previous lines of antineoplastic therapy, relapse or refractory after last therapy and previous autologous hematopoietic stem-cell transplantation (aHSCT).

To balance the average baseline characteristics between tisa-cel and conventional treatment groups, we assigned a statistical weight for each patient in conventional treatment group based on the variables adjusted in the MAIC [10]. After assigning the weights, OS was compared using the statistical tests with propensity score weights; (1) log-rank test to compare observed KM curve of JULIET with the weighted KM curve of conventional therapy group; (2) weighted Cox proportional hazard model to estimate adjusted hazard ratio (aHR) with corresponding 95% confidence interval (CI). This allowed the comparison between tisa-cel and conventional treatment in a balanced patient population. Comparisons without weighting were also reported.

As an ad-hoc sensitivity analysis, we attempted to align treatment period of the conventional treatment group to tisa-cel group, which was between 2015 and 2017, to account for potential impact of changing treatment landscapes across time on the study outcome. We created a modified conventional treatment group by including only those who received treatment between 2015 and 2017 in the SMC-LCS to compare OS with tisa-cel group.

All analyses were conducted using SAS 9.4 software (SAS Institute Inc., Cary, NC, USA), and statistical significance was considered at a level of 0.05.

The institutional review board of Samsung Medical Center approved the study (IRB No. SMC 2022-03-087-001); the board waived the requirement for obtaining informed consent as this study used anonymized administrative data.

## Results

**Fig. 1:**
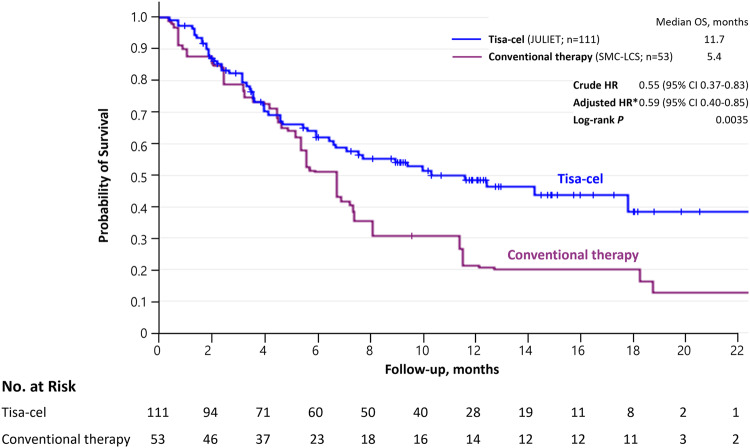
Weighted Kaplan–Meier curves for overall survival in patients treated with Tisagenlecleucel (JULIET) and conventional treatments (SMC-LCS). *Matching-adjusted indirect comparison(MAIC)-weighted hazard ratio based on age, ECOG performance, disease stage at study entry, number of previous lines of antineoplastic therapy, relapse after last therapy, refractory diffuse large B-cell lymphoma, and previous aHSCT. Abbreviation: CI confidence interval, HR hazard ratio, OS overall survival.

Of 2321 patients in SMC-LCS, 244 (10.5%) were with r/r DLBCL (Table S2). After applying eligibility criteria, 111 in JULIET FAS and 53 in the conventional treatment group were included in the analysis (Fig. S1). Median age was 56 vs. 55 years for tisa-cel vs. conventional treatment group, respectively. Before weighting, most conventional treatment group had higher proportion of patients with relapse after last therapy (73.6% vs. 45.0%), but had lower proportion of patients with refractory DLBCL (26.4% vs. 55.0%) and received prior aHSCT (20.8% vs. 48.6%), compared with the tisa-cel group (Table 1). After weighting, the differences were reduced slightly but baseline characteristics did not achieve balance. The effect sample size of conventional treatment group after weighting was *N* = 68.7, a 29.6% increase from the original sample size. Figure 1 illustrates the KM curves for OS. Tisa-cel was associated with a lower hazard of death in both before (crude HR 0.55 [95% CI 0.37–0.83]) and after adjustment (aHR 0.59 [0.40–0.85]) and median OS of 11.7 months vs. 5.4 months (conventional treatment). By the end of study period, all-cause mortality rates were 48.6% (54/111) for tisa-cel vs. 88.7% (47/53) for conventional treatment groups.

**Table 1:** Characteristics of the patients in the JULIET and SMC-LCS at baseline, before and after weighting.

	Before weighting			After weighting^a^		
	JULIET (Tisagenlec-leucel)	SMC-LCS (Conventional Therapies)	aSD	JULIET (Tisagenlec-leucel)	SMC-LCS (Conventional Therapies)	aSD
	*N* = 111	*N* = 53		*N* = 111	*N* = 68.7	
Median age (range), year	56 (22–76)	55 (25–76)		56 (22–76)	55 (25–76)	
Age ≥ 65 year	25 (23)	13 (24.5)	0.05	25 (23)	20.5 (29.8)	0.17
ECOG performance, no. (%)						
0	61 (55)	26 (49.1)	0.12	61 (55)	43.4 (63.1)	0.17
1	50 (45)	27 (50.9)	0.12	50 (45)	25.4 (36.9)	0.17
Disease stage at study entry, no. (%)						
Stage I	8 (7.2)	3 (5.7)	0.06	8 (7.2)	5.9 (8.6)	0.05
Stage II	19 (17.1)	4 (7.5)	0.29	19 (17.1)	9.3 (13.5)	0.10
Stage III	22 (19.8)	11 (20.8)	0.02	22 (19.8)	17.8 (25.9)	0.15
Stage IV	62 (55.9)	35 (66.0)	0.21	62 (55.9)	35.8 (52.1)	0.08
Bone marrow involvement at study entry, no. (%)	8 (7.2)	6 (11.3)	0.14	8 (7.2)	6.8 (10.0)	0.10
Missing data	0 (0)	22 (41.5)	1.19	0 (0)	34.6 (50.3)	1.42
Diagnosis on central histological review, no. (%)						
Diffuse large B-cell lymphoma, not otherwise specified	88 (79.3)	53 (100)	0.72	88 (79.3)	68.7 (100)	0.72
Transformed follicular lymphoma	21 (18.9)	0 (0)	0.68	21 (18.9)	0 (0)	0.68
Other	2 (1.8)	0 (0)	0.19	2 (1.8)	0 (0)	0.19
Double- or triple-hit rearrangement: MYC plus BCL2, BCL6, or both, no./total no. (%)	19/70 (27.1)	1/45 (2.2)	0.75	19/70 (27.1)	0/51.35 (0)	0.86
Cell of origin of cancer, no. (%)						
Germinal center B-cell type	63 (56.8)	1 (1.9)	1.50	63 (56.8)	0.5 (0.7)	1.58
Non-germinal center B-cell type	45 (40.5)	15 (28.3)	0.26	45 (40.5)	18.5 (26.9)	0.29
Missing data	3 (2.7)	37 (69.8)	1.95	3 (2.7)	49.7 (72.3)	2.07
No. of previous lines of antineoplastic therapy, no. (%)						
1	5 (4.5)	0 (0)	0.31	5 (4.5)	0 (0.0)	0.31
2	49 (44.1)	45 (84.9)	0.94	49 (44.1)	44.0 (64.0)	0.41
3	34 (30.6)	8 (15.1)	0.34	34 (30.6)	24.7 (36.0)	0.15
4–6	23 (20.7)	0 (0)	0.72	23 (20.7)	0 (0.0)	0.72
Relapse after last therapy, no. (%)	50 (45.0)	39 (73.6)	0.61	50 (45.0)	35.8 (52.1)	0.14
Refractory diffuse large B-cell lymphoma, no. (%)	61 (55.0)	14 (26.4)	0.61	61 (55.0)	32.9 (47.9)	0.14
Previous aHSCT, no. (%)	54 (48.6)	11 (20.8)	0.61	54 (48.6)	24.7 (36.0)	0.26

Abbreviation: *aSD* absolute standardized mean difference, *ECOG* Eastern Cooperative Oncology Group, *aHSCT* autologous hematopoietic stem cell transplantation.

^a^Matching-adjusted indirect comparison(MAIC) weights based on age, ECOG performance, disease stage at study entry, number of previous lines of antineoplastic therapy, relapse after last therapy, refractory diffuse large B-cell lymphoma, and previous aHSCT.

In the ad-hoc sensitivity analysis, 21 patients in the modified conventional treatment group were compared with tisa-cel group (Table S3). Their median OS was 5.7 months, corresponding to aHR of 0.52 (95% CI 0.23–1.16; *p* = 0.0937) for OS in tisa-cel vs. conventional treatment (Fig. S2).

## Discussion

Currently, tisa-cel is recommended as the third line or subsequent therapy for r/r DLBCL, however, the real-world evidence comparing the efficacy of tisa-cel against that of the standard of care for r/r DLBCL is scarce. In this study, published summary data of JULIET study and IPD of SMC-LCS were indirectly compared to demonstrate the comparative effectiveness of tisa-cel in improving survival of patients with r/r DLBCL. We found that, among relapsed or refractory patients after second-line therapy, the novel CAR-T therapy led to 41% reduction in the risk of death compared with South Korean patients who received conventional third-line chemotherapy and above. While promising, this finding needs to be interpreted with caution, considering for the impact of potential bias from the remaining imbalances in the baseline characteristics between the two groups after applying MAIC.

The magnitude of OS benefit with tisa-cel observed in our study was largely consistent with the findings from previous study using IPD of JULIET and CORAL (Collaborative Trial in Relapsed Aggressive Lymphoma; NCT00137995) studies [4, 11]. After applying fine stratification weights based on propensity score, the study reported OS of 12.5 months and 4.4 months, corresponding to HR of 0.44 (95% CI, 0.32–0.59; *p* < 0.001) for tisa-cel vs CORAL arm, respectively.

Indeed, choice of comparator arm would have major influence on the estimand of tisa-cel. In a study submitted to the European Medicines Agency (EMA), IPD of JULIET and published summary data of SCHOLAR-1 (International, multicohort retrospective non-Hodgkin lymphoma research) studies were compared using MAIC. Patients from SCHOLAR-1 had relatively longer median OS of 6.3 months, compared with the 5.7 and 4.4 months in SMC-LCS and CORAL studies, respectively, resulting in HR for OS of 0.68 (95% CI, 0.48–0.96) [12]. Along with the existing data, our study further supports improved patient survival with tisa-cel therapy across comparison with various external comparator cohorts.

The main strength of this study is the use of real-world data as an external comparator group to demonstrate the comparative effectiveness of tisa-cel therapy. The CORAL study used for comparison with JULIET study was a historical control arm of randomized controlled trial, hence less likely to represent real-world patients with DLBCL. In contrast, SMC-LCS represents real-world patients who had been treated at a tertiary hospital in South Korea. However, limitations related to non-randomized analysis need to be considered in interpreting this study’s findings. First, despite the use of MAIC in attempt to match baseline characteristics of eligible patients from SMC-LCS to summary characteristics of JULIET study, such method could not provide the same level of evidence with the randomization. The remaining imbalances in some prognostic factors between the two groups can exaggerate or understate the comparative effectiveness of tisa-cel. However, higher prevalence of ECOG score of 1, gene (i.e., double or triple hit) rearrangement and previous aHSCT, which are poor prognostic factors in B-cell lymphoma [13], in tisa-cel group suggest exaggeration of the observed benefit to be less likely. Second, potential bias arising from comparison with non-contemporaneous external control needs to be considered. However, the consistent finding from sensitivity analysis aligning the treatment period of the SMC-LCS to JULIET study suggests such bias to be minimal. Third, we could not rule out the possibility of potential selection bias and overestimation of comparative effectiveness estimates caused by the discrepancy in treatment process between the tisa-cel and conventional treatment; tisa-cel requires a manufacturing process that can introduce prolonged period from enrollment to infusion in FAS population. Fourth, Outcomes other than OS reported in the JULIET study were not available in the SMC-LCS as it is a health data collected during routine care and did not use standardized criteria of JULIET study in measuring responses to a given treatment. Lastly, JULIET study is an international study, whereas SMC-LCS is real-world control arm from South Korea; there were several contextual factors that could not be controlled for in the study design. Nonetheless, this study adds to the existing evidence by showing tisa-cel is expected to improve OS in patients with r/r DLBCL among Korean population.

### Supplementary information

Supplementary Material

## Data Availability

No additional data available. Data cannot be made publicly available for ethical and legal reasons, that is public availability would compromise patient confidentiality as data tables list single counts of individuals rather than aggregated data.
