# Supplementary material for: Comparison of tisagenlecleucel with conventional treatments for relapsed/refractory diffuse large B-cell lymphomas: a retrospective external comparator study
Source: Blood Cancer J. 2023 Aug 18;13(1):123. doi: 10.1038/s41408-023-00889-5 (PMC10435565; doi:10.1038/s41408-023-00889-5)
Supplement: Supplementary file 1 — Supplementary Material [file 41408_2023_889_MOESM1_ESM.docx]

**Supplementary Material**

Comparison of tisagenlecleucel with conventional treatments for relapsed/refractory diffuse large B-cell lymphomas: a retrospective external comparator study

**Contents**

**List of supplementary tables**

**Table S1. Implementation of JULIET trial eligibility criteria in SMC-LCS registry data to create external comparator cohort**………………………………………………………..2

**Table S2. Characteristics for the all patients with r/r DLBCL in the SMC-LCS at initial diagnosis and index treatment**……………………………………………………………….4

**Table S3. Characteristics of the patients in the JULIET and modified SMC-LCS (2015-2017) at baseline, before and after weighting**……………………………………………….5

**List of supplementary figures**

**Figure S1. Sample selection for the comparator population from SMC-LCS**……………..7

**Figure S2. Weighted Kaplan-Meier curves for overall survival in patients treated with Tisagenlecleucel (JULIET) and conventional therapy (modified SMC-LCS [2015-2017])**……………………………………………………………………………………….…8

**Table S1.** Implementation of JULIET trial eligibility criteria in SMC-LCS registry data to create external comparator cohort

| **Criteria** | | **Implementation in SMC-LCS registry data to create external comparator cohort** |
| --- | --- | --- |
| **Inclusion criteria** | | |
| Aged 18 years or older | | Same |
| Histologically confirmed DLBCL at last relapse(by central pathology review before enrolment. | | Same |
| Measurable disease at time of enrollment | | N/A |
| Life expectancy ≥12 weeks | | N/A |
| Eastern Cooperative Oncology Group (ECOG) performance status that is either 0 or 1 at screening | | Same, but including patients with missing data |
| Adequate organ function: | | N/A |
|  | Renal function defined as:  - A serum creatinine of ≤1.5 x Upper Limit of Normal ULN OR  - Estimated Glomerular Filtration Rate (eGFR) ≥ 60 mL/min/1.73 m2 |  |
|  | Liver function defined as:  - Alanine Aminotransferase (ALT) ≤ 5 times the Upper Limit of Normal (ULN) for age  - Bilirubin ≤ 2.0 mg/dl with the exception of patients with Gilbert-Meulengracht syndrome; patients with Gilbert-Meulengracht syndrome may be included if their total bilirubin is ≤ 3.0 x ULN and direct bilirubin ≤ 1.5 x ULN |  |
|  | Must have a minimum level of pulmonary reserve defined as ≤ Grade 1 dyspnea and pulse oxygenation > 91% on room air |  |
|  | Hemodynamically stable and Left Ventricle Ejection Fraction (LVEF) ≥ 45% confirmed by echocardiogram or Multigated Radionuclide Angiography (MUGA) |  |
|  | Adequate bone marrow reserve without transfusions defined as:  - Absolute neutrophil count (ANC) > 1.000/mm3  - Absolute lymphocyte count (ALC) ≥ 300/mm3  - Platelets ≥ 50.000//mm3  - Hemoglobin > 8.0 g/dl |  |
| Women of child-bearing potential (defined as all women physiologically capable of becoming pregnant) and all male participants must agree to use highly effective methods of contraception for at least 12 months following CTL019 infusion and until CAR T cells are no longer present by PCR on two consecutive tests | | N/A |
| **Exclusion criteria** | | |
| Prior treatment with any prior anti-CD19/anti-CD3 therapy, or any other anti-CD19 therapy | | N/A |
| Treatment with any prior gene therapy product | | N/A |
| Active Central Nervous System (CNS) involvement by malignancy | | Same, but including patients with missing data |
| Prior allogeneic HSCT | | Same |
| Eligible for and consenting to ASCT | | N/A |
| Chemotherapy other than lymphodepleting chemotherapy within 2 weeks of infusion | | N/A |
| The following medications are excluded:  - Steroids: Therapeutic doses of steroids must be stopped > 72 hours prior to CTL019 infusion. However, the following physiological replacement doses of steroids are allowed: < 6 - 12 mg/m2/day hydrocortisone or equivalent  - Immunosuppression: Any immunosuppressive medication must be stopped ≥ 4 weeks prior to enrollment  - Antiproliferative therapies other than lymphodepleting chemotherapy within two weeks of infusion  - Antibody use including anti-CD20 therapy within 4 weeks prior to infusion or 5 half-lives of the respected antibody, whichever is longer  - CNS disease prophylaxis must be stopped > 1 week prior to CTL019 infusion (e.g. intrathecal methotrexate) | | N/A |
| Prior radiation therapy within 2 weeks of infusion | | N/A |
| Active replication of or prior infection with hepatitis B or active hepatitis C( HCV RNA positive ) | | Same, but including patients with missing data |
| HIV positive patients | | Same, but including patients with missing data |
| Uncontrolled acute life threatening bacterial, viral or fungal infection (e.g. blood culture positive ≤ 72 hours prior to infusion) | | N/A |
| Unstable angina and/or myocardial infarction within 6 months prior to screening | | N/A |
| Previous or concurrent malignancy with the following exceptions:  - Adequately treated basal cell or squamous cell carcinoma (adequate wound healing is required prior to study entry)  - In situ carcinoma of the cervix or breast, treated curatively and without evidence of recurrence for at least 3 years prior to the study  - A primary malignancy which has been completely resected and in complete remission for ≥ 5 years | | Previous or concurrent malignancy any time before the index date |
| Investigational medicinal product within the last 30 days prior to screening | | N/A |
| Pregnant or nursing (lactating) women | | N/A |
| Intolerance to the excipients of the CTL019 cell product | | N/A |
| Cardiac arrhythmia not controlled with medical management | | N/A |
| Patients on oral anticoagulation therapy | | N/A |
| Prior treatment with any adoptive T cell therapy | | N/A |
| Patients with active neurological auto immune or inflammatory disorders(e.g. Guillain Barre Syndrome, Amyptrophic Lateral Sclerosis) | | N/A |

Table S2. Characteristics for the all patients with r/r DLBCL in the SMC-LCS at initial diagnosis and index treatment

|  | DLBCL patients  in SMC-LCS  (N=244) |  | DLBCL patients  with index treatment  (N=225) | |
| --- | --- | --- | --- | --- |
|  | At initial diagnosis |  | At initial diagnosis | At index treatment |
| Median age (range), yr | 56 (16-85) |  | 57 (16-85) | 57 (16-85) |
| Age ≥ 65 yr, no. (%) | 70 (29) |  | 70 (31) | 70 (31) |
| Sex, no. (%) |  |  |  |  |
| Male | 134 (55) |  | 129 (57) | 129 (57) |
| Female | 110 (45) |  | 96 (43) | 96 (43) |
| ECOG performance status, no. (%) | |  |  |  |
| 0 | 76 (31) |  | 68 (30) | 64 (28) |
| 1 | 127 (52) |  | 119 (53) | 55 (24) |
| ≥ 2 | 38 (16) |  | 35 (16) | 17 (8) |
| Missing data | 3 (1) |  | 3 (1) | 89 (40) |
| Disease stage, no. (%) |  |  |  |  |
| Stage Ⅰ | 32 (13) |  | 30 (13) | 18 (8) |
| Stage Ⅱ | 47 (19) |  | 42 (19) | 28 (12) |
| Stage Ⅲ | 35 (14) |  | 33 (15) | 32 (14) |
| Stage Ⅳ | 129 (53) |  | 119 (53) | 121 (54) |
| Missing data | 1 (1) |  | 1 (0) | 26 (12) |
| Number of extranodal sites, no. (%) | |  |  |  |
| 0 | 29 (12) |  | 25 (11) | 20 (9) |
| 1 | 87 (36) |  | 83 (37) | 71 (31) |
| ≥ 2 | 128 (52) |  | 117 (52) | 107 (48) |
| Missing data | 0 (0) |  | 0 (0) | 27 (12) |
| Bone marrow involvement, no. (%) | 59 (24) |  | 57 (25) | 36 (16) |
| Missing data | 0 (0) |  | 0 (0) | 91 (40) |
| Double- or triple-hit rearrangement, no. (%) |  |  |  |  |
| Double hits | 4 (2) |  | 4 (2) | 7 (3) |
| Triple hits | 0 (0) |  | 0 (0) | 4 (2) |
| Other | 8 (3) |  | 8 (4) | 3 (1) |
| Missing data | 0 (0) |  | 0 (0) | 52 (23) |
| Cell of origin of cancer, no. (%) |  |  |  |  |
| Germinal center B-cell type | 39 (16) |  | 34 (15) | 14 (6) |
| Non-germinal center B-cell type | 77 (32) |  | 72 (32) | 52 (23) |
| Missing data | 128 (52) |  | 119 (53) | 159 (71) |
| CNS Involvement, no. (%) | 13 (5) |  | 12 (5) | 43 (19) |
| Missing data | 0 (0) |  | 0 (0) | 25 (11) |
| Abbreviation: ECOG, Eastern Cooperative Oncology Group; CNS, Central Nervous System. | | | | |

Table S3. Characteristics of the patients in the JULIET and modified SMC-LCS (2015-2017) at baseline, before and after weighting

|  | Before weighting | | | After weighting^†^ | | |  |
| --- | --- | --- | --- | --- | --- | --- | --- |
|  | JULIET  (Tisagenlec-leucel) | SMC-LCS  (Conventional Therapies) | aSD | JULIET  (Tisagenlec-  leucel) | SMC-LCS  (Conventional Therapies) | aSD | |
|  | N = 111 | N = 21 |  | N = 111 | N = 8.1 |  | |
| Median age (range), year | 56 (22-76) | 57 (32-75) |  | 56 (22-76) | 59 (32-75) |  | |
| Age ≥ 65 year | 25 (23) | 6 (28.6) | 0.14 | 25 (23) | 3.5 (43.5) | 0.46 | |
| ECOG performance, no. (%) |  |  |  |  |  |  | |
| 0 | 61 (55) | 10 (47.6) | 0.15 | 61 (55) | 4.6 (56.9) | 0.04 | |
| 1 | 50 (45) | 11 (52.4) | 0.15 | 50 (45) | 3.5 (43.1) | 0.04 | |
| Disease stage at study entry, no. (%) |  |  |  |  |  |  | |
| Stage I | 8 (7.2) | 2 (9.5) | 0.08 | 8 (7.2) | 1.0 (12.5) | 0.18 | |
| Stage II | 19 (17.1) | 1 (4.8) | 0.40 | 19 (17.1) | 1 (12.3) | 0.14 | |
| Stage III | 22 (19.8) | 3 (14.3) | 0.15 | 22 (19.8) | 0.3 (3.8) | 0.51 | |
| Stage IV | 62 (55.9) | 15 (71.4) | 0.33 | 62 (55.9) | 5.8 (71.2) | 0.32 | |
| Bone marrow involvement at study entry, no. (%) | 8 (7.2) | 2 (9.5) | 0.08 | 8 (7.2) | 0.5 (6.0) | 0.05 | |
| Missing data | 0 (0) | 9 (42.9) | 1.23 | 0 (0) | 4.7 (57.5) | 1.65 | |
| Diagnosis on central histological review, no. (%) |  |  |  |  |  |  | |
| Diffuse large B-cell lymphoma, not otherwise specified | 88 (79.3) | 21 (100) | 0.72 | 88 (79.3) | 8.1 (100) | 0.72 | |
| Transformed follicular lymphoma | 21 (18.9) | 0 (0) | 0.68 | 21 (18.9) | 0 (0) | 0.68 | |
| Other | 2 (1.8) | 0 (0) | 0.19 | 2 (1.8) | 0 (0) | 0.19 | |
| Double- or triple-hit rearrangement: MYC plus BCL2, BCL6, or both, no./total no. (%) | 19/70 (27.1) | 1/19 (5.3) | 0.75 | 19/70 (27.1) | 0/8.1 (0) | 0.86 | |
| Cell of origin of cancer, no. (%) |  |  |  |  |  |  | |
| Germinal center B-cell type | 63 (56.8) | 1 (4.8) | 1.36 | 63 (56.8) | 0.2 (2.0) | 1.51 | |
| Non-germinal center B-cell type | 45 (40.5) | 10 (47.6) | 0.14 | 45 (40.5) | 2.3 (28.1) | 0.26 | |
| Missing data | 3 (2.7) | 10 (47.6) | 1.21 | 3 (2.7) | 5.7 (69.9) | 1.95 | |
| No. of previous lines of antineoplastic therapy, no. (%) |  |  |  |  |  |  | |
| 1 | 5 (4.5) | 0 (0) | 0.31 | 5 (4.5) | 0 (0) | 0.31 | |
| 2 | 49 (44.1) | 19 (90.5) | 1.14 | 49 (44.1) | 6.1 (75.3) | 0.67 | |
| 3 | 32 (28.8) | 2 (9.5) | 0.51 | 32 (28.8) | 2 (24.7) | 0.09 | |
| 4-6 | 23 (20.7) | 0 (0) | 0.72 | 23 (20.7) | 0 (0) | 0.72 | |
| Relapse after last therapy, no. (%) | 50 (45.0) | 19 (90.5) | 1.11 | 50 (45.0) | 5.4 (66.7) | 0.45 | |
| Refractory diffuse large B-cell lymphoma, no. (%) | 61 (55.0) | 2 (9.5) | 1.11 | 61 (55.0) | 2.7 (33.3) | 0.45 | |
| Previous aHSCT, no. (%) | 54 (48.6) | 3 (14.3) | 0.80 | 54 (48.6) | 2 (24.7) | 0.51 | |
| Abbreviation: aSD, absolute standardized mean difference; ECOG, Eastern Cooperative Oncology Group; aHSCT, autologous hematopoietic stem cell transplantation.  ^†^Matching-adjusted indirect comparison(MAIC) weights based on age, ECOG performance, disease stage at study entry, number of previous lines of antineoplastic therapy, relapse after last therapy, refractory diffuse large B-cell lymphoma, and previous aHSCT. | | | | | | |  |

| Samsung Medical Center Lymphoma Cohort Study (SMC-LCS) (N = 2,321)  1) Prospective Cohort Study Ⅰ (NCT#00822731) (N = 953)  2) Prospective Cohort Study Ⅱ (NCT#01877109) (N = 1,368) | | | |
| --- | --- | --- | --- |
|  |  | | |
| Histologically confirmed Diffuse Large B-Cell Lymphoma (DLBCL) (N = 949)  1) Prospective Cohort Study Ⅰ (NCT#00822731) (N = 390)  2) Prospective Cohort Study Ⅱ (NCT#01877109) (N = 559) | | | |
|  |  | | |
| Patients with DLBCL who received 1L therapy (N = 244) | | | |
|  |  | |  |
| Relapsed or refractory disease after ≥ 2 lines of chemotherapy,  either having failed autoHCT on the index treatment (N = 225) | | | |
|  |  | | |
| Patients aged ≥ 18 years at initial diagnosis (N = 225) | | | |
|  |  | | |
| Patients who received 3L+ therapy (N = 180) | | | |
|  |  | | |
| ECOG performance status of 0 or 1 on the index treatment (N = 168) | | | |
|  |  | |  |
|  |  | | Excluded (N=70)  27 Patients with CNS involvement  0 Patients who previously received alloHCT  6 Previous or concurrent a primary malignancy  37 Prior infection with hepatitis or HIV positive |
|  |  | |  |
|  |  | |  |
| **Eligible patients (N = 98)** | | | |
|  |  |  | |
|  |  | Excluded (N=)  5 Patients who were outside the age range of JULIET study (22–76 years)  40 Patients without information on ECOG and disease stage | |
|  |  |  |  |
|  |  |  | |
| **Cohort for applying MAIC (N = 53)** | | | |

Figure S1. Sample selection for the comparator population from SMC-LCS

Abbreviation: alloHSCT, allogeneic hematopoietic stem cell transplantation; aHSCT, autologous hematopoietic stem cell transplantation; DLBCL, diffuse large B-cell lymphoma; ECOG, Eastern Cooperative Oncology Group; MAIC, matching-adjusted indirect comparison.


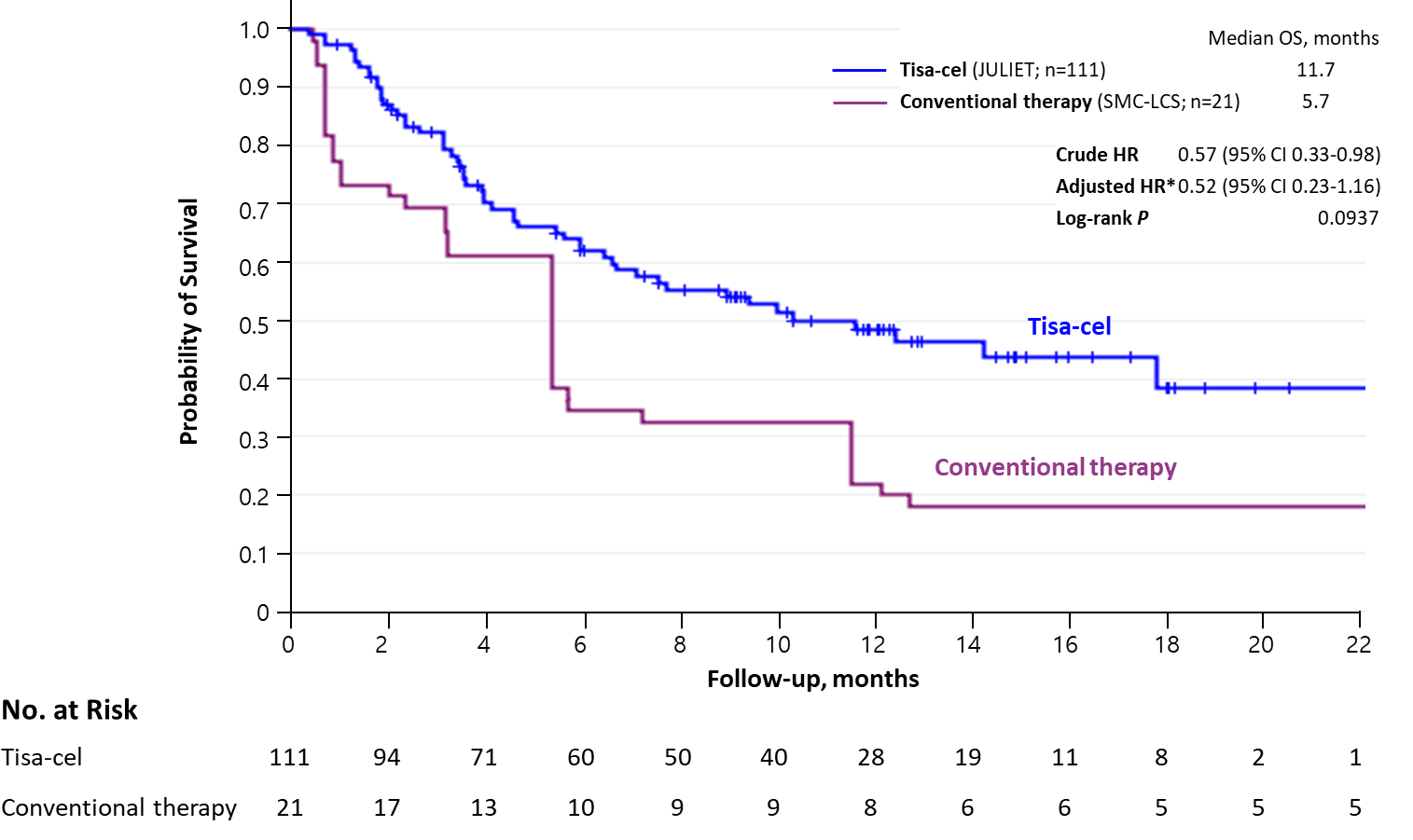


**Figure S2.** Weighted Kaplan-Meier curves for overall survival in patients treated with Tisagenlecleucel (JULIET) and conventional therapy (modified SMC-LCS [2015-2017])

*Matching-adjusted indirect comparison(MAIC)-weighted hazard ratio based on age, ECOG performance, disease stage at study entry, number of previous lines of antineoplastic therapy, relapse after last therapy, refractory diffuse large B-cell lymphoma, and previous aHSCT.

Abbreviation: CI, confidence interval; HR, hazard ratio; OS, overall survival.
